# Effects of ensiling processes and antioxidants on fatty acid concentrations and compositions in corn silages

**DOI:** 10.1186/2049-1891-4-48

**Published:** 2013-12-04

**Authors:** Liying Han, He Zhou

**Affiliations:** 1 College of Animal Science and Technology, China Agricultural University, Yuanmingyuan West Road 2#, Haidian District Beijing, PRC, 100193, China

**Keywords:** Antioxidant, Fatty acid, Silage

## Abstract

**Background:**

Corn silage is the main dietary component used for ruminant breeding in China and is an important dietary source of fatty acids for these animals. However, little is known regarding effective means to protect the fatty acid (FA) contents in silages. In this study, we examined the changes in FA contents and compositions during corn ensiling and screened several antioxidants for their inhibition of lipid oxidation during corn ensiling.

**Methods:**

We conducted two different experiments. In Experiment 1, corn was ensiled in 30 polyethylene bottles (bottle volume: 1 L, silage density: 600 g/dm^3^) and three bottles were opened at 0.5 d, 1 d, 1.5 d, 2 d, 2.5 d, 3 d, 5 d, 7 d, 14 d, and 28 d after ensiling. In Experiment 2, corn was treated with various antioxidants: (1) No additives (CK); (2) BHA (Butylated hydroxyanisole); (3) TBHQ (Tertiary butyl hydroquinone); (4) TPP (Tea polyphenols); and (5) VE (Vitamin E). These treatments were applied at 50 mg/kg and 100 mg/kg of fresh weight with each treatment replicated 3 times.

**Results:**

During ensiling in Experiment 1, saturated fatty acids (SFA; C16:0 and C18:0) and malondialdehyde (MDA) contents tended to increase, whereas unsaturated fatty acids (UFA; C18:1, C18:2 and C18:3) tended to decrease. However, these changes were only significant on the first 2 days of ensiling. In Experiment 2, all of the antioxidants tested affected the total FA contents and those of unsaturated fatty acids (C18:1, C18:2 and C18:3) and MDA. The effects of TBHQ and TPP were greater than those of the other antioxidants.

**Conclusions:**

The reduced total FA contents in corn silages were due to unsaturated fatty acids’ oxidation during the early stages of ensiling. Adding an antioxidant could prevent fatty acids’ oxidation in corn silages.

## Background

Corn silage is the main dietary component used for ruminant breeding in China and is an important dietary source of fatty acids for these animals. However, the ensiling process may reduce the positive effects of herbage lipids on the fatty acid (FA) composition of milk due to oxidation during the period between plant cutting and ensiling [1,2]. During harvesting and the early stages of ensiling, enzymatic hydrolysis of triacylglycerols yield free fatty acids (FFAs) from damaged tissues after cutting and non-esterified polyunsaturated fatty acids (PUFA) from damaged membranes are rapidly converted to hydroperoxy PUFA by the actions of lipoxygenases (LOX) [3]. The most common substrates for plant lipoxygenases are linolenic and linoleic acids. These are abundant in plant membranes and can be further broken down into aldehydes and ketones [4], which may affect feed preferences, palatability, and ingestion by animals [5,6].

Numerous studies have been done on chemically characterizing silages and their nutritional value in order to obtain high quality silages [7-9]. Dewhurst et al. [10] concluded that plant species and cutting intervals affected the FA compositions of grasses. The effects of additives, such as formalin, formic acid, inoculants, and enzymes, on the FA compositions of grass silages have also been investigated [11-14], although these results suggested that they only had minimal effects on the FA contents. Lourenco et al. [15] and Lee et al. [16] attributed reduced lipid oxidation in red or white clover silages to their high polyphenol oxidase (PPO) contents. However, due to wilting and ensiling, ensiled forages contain fewer antioxidants as compared to fresh pasture forage [13,17]. Some antioxidant phenolic compounds have been widely used to inhibit lipid oxidation in the food industry. Thus, it might be possible to use these as silo lipid oxidation inhibitors.

Thus, in this study, we examined the changes in FA contents and compositions during corn ensiling and screened several antioxidants for their inhibition of lipid oxidation during corn ensiling.

## Methods

### Corn and ensiling

We used corn for our experiments (*Zea mays* L., JKN928), which was sown on April 25, 2012. The temperature range and total precipitation during the growing season were 16.2–27.4°C and 94.5 mm, respectively. We selected a high cutting height for corn (1 m above ground) in order to increase the FA contents during ensiling. To determine their compositions, 10 plants from 10 randomly selected sites were sampled, chopped, and stored at -80°C. Corn, including the ear, was harvested at the one-half milk line stage (August 5, 2012) and chopped into 10 mm lengths using a conventional forage harvester. Then, we used two different experiments.

In Experiment 1, corn was ensiled in 30 polyethylene bottles (bottle volume: 1 L, silage density: 600 g/dm^3^) in the dark at 25 ± 2°C. Each treatment was replicated 3 times. Three bottles were opened after having been ensiled for 0.5 d, 1 d, 1.5 d, 2 d, 2.5d, 3 d, 5 d, 7 d, 14d, and 28 d. About 400 g samples from each bottle were removed and vacuum packed at –18°C to determine the fermentation quality, FA contents and compositions, and malondialdehyde (MDA) contents.

In Experiment 2, corn was divided into equal portions for different treatments. These treatments were: (1) No additives (CK); (2) Butylated hydroxyanisole (BHA, synthetic antioxidant); (3) Tertiary butyl hydroquinone (TBHQ, synthetic antioxidant); (4) Tea polyphenols (TPP, natural antioxidant); and (5) Vitamin E (VE, natural antioxidant). These treatments were applied at 50 mg/kg and 100 mg/kg of fresh weight. All antioxidants were purchased from Beijing Sky Bamboo Bird Food Additives Co., Ltd. (Beijing, China). The antioxidants were diluted with distilled water to obtain the designated application concentrations and sprayed onto fresh corn. For a control, the same amount of distilled water was sprayed onto corn samples. About 200 g of each treated or untreated corn sample was frozen immediately in liquid nitrogen and used to determine LOX activity.

Treated and untreated corn samples were ensiled in polyethylene bottles (bottle volume: 1 L, silage density: 600 g/dm^3^) in the dark at 25 ± 2°C for 60 d. Each treatment was replicated 3 times. The FA contents and compositions (C16:0, C18:0, C16:1, C18:1, C18:2, C18:3), and the contents of MDA, fermentation quality, dry matter (DM), water soluble carbohydrates (WSC), crude proteins (CP), neutral detergent fiber (NDF), and acid detergent fiber (ADF) in these silages were determined when the silage bottles were opened.

### Chemical analyses

Using 250 μmol/L linolenic acid as the substrate, lipoxygenase (LOX) activity was determined as the increase in absorbance at 234 nm due to the formation of conjugated dienes using a spectrophotometer over 5–10 min [18]. A sample (1 g) was diluted in 50 mmol/L Na phosphate buffer (pH 7.0) and incubated for 30 min on ice with occasional vortexing. The sample was then centrifuged (10,000 rpm) at 4°C for 30 min and the supernatant was used as a crude enzyme solution. Protein concentrations in the enzyme solutions were determined using the Bradford method (Bio-Rad, Hercules, CA, USA).

To initiate the assay, 0.05 mL of an enzyme extract was mixed with 0.25 mL of substrate stock solution, followed by incubation at 30°C for 4 min. After incubation, 1 mol/L NaOH (0.7 mL) was added to stop the reaction. Hydroperoxides produced by LOX were monitored using a spectrophotometer (Thermo Electron Co., PA, USA) at 234 nm. One unit of enzyme activity was defined as an increase in absorbance of 0.001 at 234 nm per mg of protein per minute (Units/mg protein/min). Protein concentrations were determined using the Coomassie Brilliant Blue method [19].

FA compositions were determined by gas chromatography (GC) after methylation [20]. GC analyses were done using a Shimadzu GC-2010 chromatograph equipped with an Agilent chromatography column for FA methyl esters (FAME) (100 m × 0.25 mm × 0.2 μm). The temperature program was: starting temperature of 180°C for 10 min, which was then increased by 4°C/min until the temperature reached 200°C; the injector temperature was set at 250°C; and the detector temperature was 280°C. Malondialdehyde (MDA) was determined spectrophotometrically as thiobarbituric acid reactive substances (TBARS) after reaction with thiobarbituric acid (TBA) at 100°C in acidic media; the absorbance of a reaction mixture was measured at 532 nm [21].

Fermentation indices were determined using the following methods. A sample silage (20 g) was homogenized in 180 mL of distilled water for 1 min at high speed (12,000 rpm). The resulting suspension was filtered through four layers of cheese cloth and then centrifuged for 20 min at 27,500 × g, after which the pellet discarded. Supernatant samples were used for pH, lactic acid, acetic acid, propionic acid, butyric acid, and NH_3_-N analyses. pH was determined with a pH meter (PHS-3C). Lactic acid, acetic acid, propionic acid, and butyric acid were determined by HPLC (SHIMADZE-10A, Shimadze, Japan) as described [22]. The HPLC system included a Shimadzu system controller (SCL-10A) and a Shodex Rspak KC-811 S-DVB gel column (300 mm × 8 mm) at a column temperature of 50°C. The mobile phase was a solution of 3 mmol perchloric acid at a rate of 1 mL/min. The injection volume was 50 μL. A UV detector (SPD-10A) was used and analyses were made at 210 nm.

Ammonia-N (NH_3_-N) was determined by the Phenol-Hypochlorite colorimetric method as described [23]. DM was determined by oven drying at 65°C for 48 h. Crude protein (CP) was determined using the Kjeldahl method [24]. NDF and ADF were analyzed as described [25]. Water-soluble carbohydrate (WSC) was determined by the Deriaz method [26].

### Statistical analysis

Statistical comparisons were made by one-way analysis of variance followed by Duncan’s new multiple range test [27]. Results for similar treatments at different time points were compared using paired t-tests. Statistical analyses were done using SAS 9.1.3 software (SAS Institute, Cary, NC, USA). *P* < 0.05 was considered significant.

## Results

### Material compositions

Dry matter (DM) contents and the chemical compositions (WSC, CP, NDF, ADF, and FA contents and compositions) of fresh chopped whole corn plants before ensiling are shown in Table 1. The DM contents and WSC of the corn used in this study were higher than the recommended contents to ensure successful ensiling and to obtain good fermentation rates [28,29]. High CP and total FA contents and low NDF and ADF contents were found because of the high cutting height used in this study (1 m above ground). More than one half of the FA in fresh corn was C18:2.

**Table 1:** DM, WSC, CP, NDF, ADF, FA contents and compositions in fresh whole plant corn

**Items**	**N**^**1**^	**Mean**	**SD**^**2**^
DM, g/kg	10	283.3	12.6
WSC, g/kg DM	10	130.4	4.0
CP, g/kg DM	10	115.4	7.5
NDF, g/kg DM	10	384.1	4.5
ADF, g/kg DM	10	271.2	2.1
Total FA content, mg/kg DM	10	24.74	0.61
Proportion in DM, mg/kg DM			
C16:0	10	4.16	0.16
C18:0	10	1.61	0.11
C18:1	10	3.86	0.27
C18:2	10	12.97	0.14
C18:3	10	1.85	0.08
Proportion in total FA,%			
C16:0	10	16.81	1.56
C18:0	10	6.51	0.73
C18:1	10	14.87	1.03
C18:2	10	52.43	3.45
C18:3	10	7.48	0.74

^1^*N*, observation numbers.

^2^*SD*, standard deviation.

### Fatty acid changes during ensiling

The FA contents and compositions and the MDA contents during ensiling are shown in Table 2. No differences were found for DM during ensiling, whereas significant decreases in WSC and pH had occurred, as was expected. Total FA contents decreased markedly during the first two days of ensiling. Significant changes in C16:0, C18:0, C18:1, C18:2, and C18:3 compositions were also found during the first two or three days of ensiling. Saturated fatty acid (SFA; C16:0 and C18:0) compositions tended to increase, whereas unsaturated fatty acid (UFA; C18:1, C18:2, and C18:3) compositions tended to decrease. The greatest decrease in the proportion of C18:2 among total FA occurred on the 28th day of ensiling. The proportion of MDA increased as the number of days of ensiling increased, although this was only significant during the first two days.

**Table 2:** Effect of the ensiling process on total FA content and composition, MDA , DM and WSC content in corn silage

**Treatments**	**0.5d**	**1d**	**1.5d**	**2d**	**3d**	**5d**	**7d**	**14d**	**28d**	**SEM**^**1**^
pH	4.61^a^	4.25^b^	4.08^c^	3.85^d^	3.76^de^	3.73^de^	3.70^e^	3.69^e^	3.68^c^	0.06
Total FA, mg/kg DM	23.68^a^	23.04^a^	21.73^b^	19.56^c^	19.43^c^	19.45^c^	19.51^c^	19.57^c^	19.75^c^	0.76
Proportion in DM, mg/kg DM										
C16:0	4.19^c^	4.27^b^	4.36^b^	4.55^a^	4.56^a^	4.58^a^	4.59^a^	4.62^a^	4.66^a^	0.18
C18:0	1.73^c^	1.78^c^	1.88^b^	1.85^b^	1.91^a^	2.04^a^	2.02^a^	2.05^a^	2.09^a^	0.09
C18:1	3.34^a^	3.01^b^	2.99^b^	2.57^c^	2.45^c^	2.48^c^	2.46^c^	2.45^c^	2.56^c^	0.21
C18:2	11.92^a^	10.64^b^	9.74^c^	8.53^d^	8.34^d^	8.23^d^	8.18^d^	8.19^d^	8.21^d^	0.26
C18:3	1.78^a^	1.59^b^	1.44^c^	1.32^d^	1.31^d^	1.27^d^	1.23^d^	1.19^d^	1.18^d^	0.20
Proportion in total FA,%										
C16:0	17.69^d^	18.53^c^	20.06^b^	23.26^a^	23.46^a^	23.54^a^	23.52^a^	23.60^a^	23.59^a^	0.41
C18:0	7.31^d^	7.73^d^	8.65^c^	9.46^b^	9.83^a^	10.49^a^	10.35^a^	10.47^a^	10.58^a^	0.52
C18:1	14.10^a^	13.06^b^	12.84^b^	13.14^b^	12.61^b^	12.24^b^	12.61^b^	12.52^b^	12.96^b^	0.35
C18:2	50.33^a^	46.18^b^	44.82^c^	43.61^d^	42.92^d^	42.31^d^	41.93^d^	41.84^d^	41.56^d^	0.62
C18:3	7.52^a^	6.90^b^	6.63^b^	6.74^b^	6.74^b^	6.53^b^	6.30^b^	6.08^b^	5.97^b^	0.24
MDA, μmol/g FW	35.12^d^	47.33^c^	60.82^b^	72.11^a^	72.18^a^	73.39^a^	73.69^a^	74.60^a^	74.89^a^	0.52
DM, g/kg FW	28.83	28.45	27.72	27.69	28.71	28.16	29.80	28.00	27.72	1.69
WSC, g/kg DM	9.93^a^	9.62^a^	9.24^a^	6.73^b^	6.71^b^	5.27^bc^	4.76^c^	2.29^d^	2.01^d^	0.86

^a,b,c,d,e^Within a row, means without a common superscript differ (*P* < 0.05).

^1^*SEM*, standard error mean.

### Lipoxygenase (LOX) activity

LOX activity results are shown in Table 3. The LOX activities of treated corn were lower than that of the control. LOX activity decreased as the concentration of additives increased for most treatments used. However, LOX activities were not detected when the added concentrations of TPP and BTHQ were 100 mg/kg.

**Table 3:** Effect of different antioxident additives on the LOX activity in corn silage

**Additives**	**Amount**	**LOX activity,%**
BHA	0, Ck^1^	100^a^
	50 mg/kg	46.32^b^
	100 mg/kg	23.71^c^
SE^2^		9.15
		
TBHQ	0, Ck	100^a^
	50 mg/kg	26.15^b^
	100 mg/kg	0.00^c^
SE		8.47
		
TPP	0, Ck	100^a^
	50 mg/kg	22.04^b^
	100 mg/kg	0.00^c^
SE		10.26
		
VE	0, Ck	100^a^
	50 mg/kg	37.69^b^
	100 mg/kg	15.33^c^
SE		9.15

^a,b,c^Within a column, means without a common superscript differ (*P* < 0.05).

^1^The LOX activity in CK was considered as 100%.

^2^*SE*, standard error.

### Effects of antioxidants

The fermentation quality and chemical compositions of the corn silages after 60 d of conservation are shown in Table 4. The pH values of all the silages when bottles were opened were < 4.0. The lactic acid (LA) contents were high, and there was little butyric acid (BA) (< 0.1 g/kg DM). All of the treatments used affected ammoniacal nitrogen/total nitrogen (NH_3_-N/TN) and WSC contents. There were no significant differences between treated and untreated silages for DM, CP, NDF, and ADF contents.

**Table 4:** Effect of different antioxidants on the formation of corn silage

**Treatments**	**pH**	**LA**	**AA**	**BA**	**NH**_**3**_**-N**	**DM**	**WSC**	**CP**	**NDF**	**ADF**
		**g/kg**	**g/kg**	**g/kg**	**g/kg**	**g/kg**	**g/kg**	**g/kg**	**g/kg**	**g/kg**
		**DM**	**DM**	**DM**	**TN**	**FW**	**DM**	**DM**	**DM**	**DM**
CK	3.75	46.3	24.1	<0.1	36.1^a^	281.7	10.3c	132.4	394.1	221.7
BHA50	3.62	47.1	23.4	<0.1	24.1^b^	285.3	13.7b	139.8	381.5	218.5
BHA100	3.60	46.9	22.5	<0.1	21.2^c^	290.7	14.3a	145.2	378.6	209.5
TBHQ50	3.71	46.4	22.0	<0.1	25.4^b^	282.4	13.2b	138.6	385.3	219.6
TBHQ100	3.70	46.3	21.3	<0.1	20.1^c^	291.1	14.1a	140.9	382.6	205.4
TPP50	3.72	48.2	24.8	<0.1	20.3^c^	292.1	14.5a	141.5	379.5	212.5
TPP100	3.70	47.8	23.9	<0.1	19.7^c^	296.9	15.2a	146.3	375.1	200.1
VE50	3.74	47.6	25.0	<0.1	21.1^c^	288.6	12.9b	136.6	388.9	214.7
VE100	3.71	47.5	24.5	<0.1	20.5^c^	294.3	14.7a	142.5	379.1	203.5
^1^SEM	0.42	4.13	2.51	--	1.33	6.72	1.72	4.12	7.25	6.57

^a,b,c^Within a column, means without a common superscript differ (*P* < 0.05).

^1^*SEM*, standard error mean.

The effects of the different antioxidants on the FA contents and compositions and the MDA contents are shown in Table 5. All of these antioxidants affected the total FA contents and treatments with TBHQ100 and TPP100 were better than the other antioxidants. There were no significant differences in saturated fatty acids (C16:0 and C18:0) between treated and untreated silages, whereas the unsaturated fatty acid (C18:1, C18:2 and C18:3) compositions of treated silages were higher than those of the control. The MDA contents in treated silages were lower than in the control.

**Table 5:** Effect of diierent antioxidants on the FA content and composition and MDA content of corn silages (mg/kg DM)

**Treatments**	**Total FA**	**C16:0**	**C18:0**	**C18:1**	**C18:2**	**C18:3**	**MDA, μmol/g FW**
CK	19.37^c^	3.34	1.62	2.89^c^	9.06^d^	1.52^c^	68.99^a^
BHA50	21.81^b^	3.36	1.65	3.06^b^	10.73^c^	1.65^b^	46.69^b^
BHA100	22.12^a^	3.29	1.56	3.15^b^	11.65^b^	1.67^b^	34.45^c^
TBHQ50	22.74^a^	3.41	1.58	2.99^b^	11.68^b^	1.79^a^	32.75^c^
TBHQ100	23.04^a^	3.30	1.51	3.11^b^	11.95^a^	1.82^a^	32.31^c^
TPP50	22.56^a^	3.35	1.55	3.22^b^	11.62^b^	1.80^a^	32.44^c^
TPP100	23.05^a^	3.22	1.50	3.56^a^	12.02^a^	1.85^a^	31.82^c^
VE50	21.95^b^	3.38	1.60	3.02^b^	10.69^c^	1.63^b^	45.68^b^
VE100	22.74^a^	3.25	1.57	3.23^b^	11.33^b^	1.72^b^	34.12^c^
^1^SEM	0.65	0.17	0.09	0.27	1.33	0.12	2.41

^a,b,c,d^Within a column, means without a common superscript differ (*P* < 0.05).

^1^*SEM*, standard error mean.

## Discussion

Corn that is ensiled along with the ear can improve the nutritional value of feed because the high WSC contents should increase bacterial activity. In this study, high WSC and CP contents and low NDF and ADF contents resulted because of the high cutting height we used. Linoleic acid (C18:2) in corn increases as the plant matures, whereas the C18:3 concentration progressively declines [30]. Thus, C18:2 corresponded to more than 50% of the total FA in this study, which was in agreement with Shingfield et al. [13].

http://Lipid oxidation depends on the activity of lipoxygenases. Lipoxygenase activity is found in a plant at all growth stages and this activity increases when a plant enters the mature and senescence stages or after tissue injury. UFA’s that are hydrolyzed from lipids are oxidized by LOX to form hydroperoxides, which are further decomposed into aldehydes and ketones. Malondialdehyde (MDA) is primarily produced via the lipoxygenase pathway of fatty acid oxidation and is widely used in food science as an index of lipid oxidation and rancidity in foods and food products [31]. Thus, for this study, MDA was determined as a product of lipid peroxidation and used as an index of lipid oxidation.

The differences in FA compositions could be related to various factors, such as plant species, cutting date, wilting, and the ensiling process [10,11,32-34]. Plant enzymes can remain functional in silages, although the activity of plant enzymes generally declines during ensiling. According to Elgersma et al [35], almost all of the total fat in fresh grass is in the form of esterified fatty acids, whereas in silage, a large proportion is in the form of free fatty acids (FFAs). These FFAs are then further oxidized by LOX.

UFAs are more susceptible to oxidation than are SFAs. Thus, the decrease in total FA contents together with the increase in SFA contents and the decreases in UFA contents on 28 d of corn ensiling were due to UFA oxidation, particularly that of C18:2. Malondialdehyde (MDA), one of the final products of lipid oxidation, increased during ensiling, which further indicated FA oxidation during ensiling. The rapid changes in FA contents and compositions mainly occurred during the first two days of ensiling in this study. This indicated that enzyme activity was higher during the first two days than during the other ensiling periods. This may have been related to the pH changes that occurred during ensiling.

A high LOX activity would be plausible in high pH silages, whereas these enzymes would be inhibited by low pH [15,36]. In addition, lipid or FA oxidation requires oxygen, whereas ensiling is a process that progresses from aerobic conditions to anaerobic conditions. Thus, LOX activity would decline as oxygen was consumed during ensiling.

Many additives, such as formalin, formic acid, inoculants, and enzymes, have been used to alter the FA contents and compositions of silages [11-14]. However, their effects were either minimal or they had no effects. Among the various methods that have recently been used to prevent lipid peroxidation, adding antioxidants has received the most attention. Most of the commonly used antioxidants are http://phenolics that can be divided into synthetic compounds and natural ingredients.

Synthetic compounds, such as BHA, BHT, and TBHQ, are chemically stable, inexpensive, and readily available. However, the safety of these synthetic antioxidants has been questioned due to their potential risks to human health [37]. Thus, there is a growing interest in natural ingredients because they are more acceptable to consumers, more palatable, stable. and improve the shelf-lives of food products. They have also shown beneficial health effects against degenerative diseases and certain cancers.

Polyphenols are known to have important protective roles during lipoperoxidation. There is considerable data for the use of polyphenols as natural antioxidants and an interest in the antioxidant properties of polyphenols from pomegranate has recently emerged [38,39]. Vitamin E is commonly added to animal and human diets because it can inhibit lipoperoxidation [40]. It is also an important nutrient that aids in stabilizing unsaturated fatty acids in milk [41,42].

Antioxidants prevent enzyme catalysis by disrupting fatty acid peroxidation chain reactions or chelating Cu and Fe ions to form stable chelation compounds [43,44]. The lipoperoxidase (LOX) activities found in this study were lower after adding antioxidants as compared to the control. These results were consistent with those in a previous report [45], which indicated that antioxidants considerably inhibited lipid oxygenation.

All of the silages in this study were of high quality due to their high lactic acid contents, low pH values, and NH_3_-N/TN contents. Furthermore, as compared to the control, all of the treatments we used affected the NH_3_-N/TN contents. The reason may have been that the antioxidants used in this study inhibited protease activity. For the same reason, the treated silages had higher CP contents than the control, although these differences were not significant. Aerobic microorganism metabolism would also be restricted because of the lower oxygen tension. Thus, the WSC contents in treated silages were higher than they were in the control on day 28 of this study. The antioxidants used in this study could not break down cellulose, so there were no significant differences between treated and untreated silages for NDF and ADF contents.

We found that all of the antioxidants we used successfully protected fatty acids. Higher total FA and UFA contents, and lower MDA contents were found in all of the treated silages. These results were consistent with the predicted loss of antioxidants in silage during ensiling [13,17]. Furthermore, TPP and TBHQ were better than BHA and VE for inhibiting FA oxidation. These results were consistent with the inhibitory effects on LOX activity shown in Table 3, which indicated that the FA losses in silages were mainly due to the effects on LOX activity and that higher FA concentrations could be obtained by inhibiting LOX activity.

## Conclusions

A reduction in total FA contents in corn silages was due to the oxidation of unsaturated fatty acids by LOX during the early stages of ensiling. However, adding an antioxidant could prevent fatty acids’ oxidation in corn silages.

## Abbreviations

DM: Dry matter; FW: Fresh weight; WSC: Water-soluble carbohydrate; CP: Crude protein; NDF: Neutral detergent fiber; ADF: Acid detergent fiber; FA: Fatty acid; MDA: Malondialdehyde; LOX: Lipoperoxidation; LA: Lactic acid; AA: Acetic acid; BA: Butyric acid; NH3-N/TN: Ammoniacal nitrogen/total nitrogen; CK: No additives; BHA: Butylated hydroxyanisole; TBHQ: Tertiary butyl hydroquinone; TPP: Teapolyphenols; VE: Vitamin E; BHA50: 50 mg butylated hydroxyanisole/kg fresh corn; BHA100: 100 mg butylated hydroxyanisole/kg fresh corn; TBHQ50: 50 mg tertiary butyl hydroquinone/kg fresh corn; TBHQ100: 100 mg tertiary butyl hydroquinone/kg fresh corn; TPP50: 50 mg teaPolyphenols /kg fresh corn; TPP100: 100 mg teaPolyphenols/kg fresh corn; VE50: 50 mg vitamin E/kg fresh corn; VE100: 100 mg vitamin E/kg fresh corn.

## Competing interests

The authors declare that they have no competing interests related to this study.

## Authors’ contributions

LYH did the chemical analyses, statistical analyses, and drafted the manuscript. HZ conceived the study, participated in its design and coordination, and helped draft the manuscript. All authors read and approved the final manuscript.
